# A Green High-Performance Thin-Layer Chromatography Method for the Determination of Caffeine in Commercial Energy Drinks and Formulations

**DOI:** 10.3390/ma15092965

**Published:** 2022-04-19

**Authors:** Ahmed I. Foudah, Faiyaz Shakeel, Mohammad A. Salkini, Sultan Alshehri, Mohammed M. Ghoneim, Prawez Alam

**Affiliations:** 1 Department of Pharmacognosy, College of Pharmacy, Prince Sattam Bin Abdulaziz University, Al-Kharj 11942, Saudi Arabia; a.foudah@psau.edu.sa (A.I.F.); m.salkini@psau.edu.sa (M.A.S.); 2 Department of Pharmaceutics, College of Pharmacy, King Saud University, Riyadh 11451, Saudi Arabia; faiyazs@fastmail.fm (F.S.); salshehri1@ksu.edu.sa (S.A.); 3 Department of Pharmacy Practice, College of Pharmacy, AlMaarefa University, Ad Diriyah 13713, Saudi Arabia; mghoneim@mcst.edu.sa

**Keywords:** AGREE scale, caffeine, energy drinks, formulations, green HPTLC, validation

## Abstract

The literature on green analytical approaches for caffeine estimation is limited. As a consequence, this study aimed to establish a reverse-phase high-performance thin-layer chromatography (HPTLC) technique for caffeine estimation in a variety of commercial energy drinks (ED) and pharmaceutical formulations that is rapid, sensitive, and green. The combination of ethanol-water (55:45 *v* *v*^−1^) was used as a mobile phase. The detection of caffeine was carried out at 275 nm. The green reverse-phase HPTLC method was linear in the concentration range of 50–800 ng band^−1^. Furthermore, the developed method for caffeine estimation was simple, quick, economical, accurate, precise, robust, sensitive, and green. The amount of caffeine in different marketed ED (ED1–ED10) was recorded in the range of 21.02–37.52 mg 100 mL^−1^ using the developed HPTLC method. However, the amount of caffeine in different commercial formulations (F1–F3) was estimated as 10.63–20.30 mg 100 mL^−1^ using the same method. The “analytical GREEnness (AGREE)” scale for the developed analytical method was predicted to be 0.80, utilizing 12 distinct components of green analytical chemistry, indicating the HPTLC approach’s excellent greener profile. Overall, the developed method for estimating caffeine in marketed ED and dosage forms was found to be reliable.

## 1. Introduction

Caffeine (1,3,7-trimethylxanthine) is a pseudo-alkaloidal compound, obtained from various plants, such as tea leaves (*Thea sinensis*), coffee beans (*Coffea arabica*), and kola nuts (*Cola nitida*) [1,2]. It has several pharmacological activities, such as central nervous system stimulant, analgesic, antipyretic, anti-inflammatory, antimigraine, and anticancer activity [1,2,3,4]. It is an active component of various painkillers and antimigraine pharmaceutical products [2] in combination with paracetamol [5]. Caffeine is also present in various commercially available energy drinks (ED) and herbal products [2]. As a result, it is critical to conduct a qualitative and quantitative investigation of caffeine in ED and herbal products. 

A wide range of analytical methods is available for caffeine estimation in commercial ED and herbal products. Several UV-based spectrometry methods have been used to quantify caffeine in commercial ED and herbal products [6,7,8,9,10]. Caffeine has also been determined in commercial ED and herbal products using a variety of high-performance liquid chromatography (HPLC) methods [11,12,13,14,15,16,17,18]. Caffeine in commercial ED has also been determined using ultra-fast liquid chromatography and ultra-performance liquid chromatography methods [19,20]. Some gas-chromatography tandem mass-spectrometry (GC-MS) methods have also been utilized for caffeine estimation in ED [21,22,23]. Caffeine measurement in commercial ED, herbal products, and pharmaceutical formulations has also been conducted using several high-performance thin-layer chromatography (HPTLC) techniques [2,9,22,24,25,26]. Furthermore, solid-phase Fourier-transform Raman spectroscopy [27], surfactant-mediated matrix-assisted laser desorption/ionization [28], microemulsion electrokinetic chromatography [29], and micellar electrokinetic chromatography [30] methods have been used for the estimation of caffeine in ED and herbal products. A single green HPTLC approach was utilized for caffeine estimation in commercial ED and herbal products using the binary combination of ethyl acetate and methanol as the green solvents [2]. However, its greenness scale was not estimated. Using ethyl-acetate-ethanol (EtOH) and EtOH-water as the mobile phases, we recently published the green normal-phase and reverse-phase HPTLC methods for the simultaneous determination of caffeine and paracetamol in allopathic formulations [31]. 

The safety and ecofriendly aspects of most of the reported analytical methods of caffeine estimation were not taken into consideration. Green HPTLC methods offer a wide range of advantages, including simplicity, economical performance, low operation costs, rapid analysis, parallel analysis of many samples, rapid detection, and reduced environmental pollution in comparison to other methods of analysis [32,33,34,35]. As a consequence, a reverse-phase HPTLC method for caffeine estimation was developed in the current study. For the assessment of greenness index of analytical methods, several ecofriendly approaches are reported [34,35,36,37,38,39]. However, the “analytical GREEnness AGREE” approach uses all twelve principles of “green analytical chemistry (GAC)” for the assessment of greener index of these techniques [38]. As a consequence, the “AGREE metric methodology” was utilized for the assessment of the developed reverse-phase HPTLC method’s green scale [38]. The investigated solvents such as EtOH and water are categorized as green solvents according to GAC principle [40,41,42]. Due to the safety and non-toxicity of EtOH and water towards the environment, these solvents are considered to be green solvents [42,43]. Accordingly, the binary mixture of EtOH and water was selected as the green solvent system in this research. The goal of the current investigation is to design and validate a rapid, sensitive, and environmentally friendly reverse-phase HPTLC method for determining caffeine in ED and herbal products. The International Conference for Harmonization (ICH)-Q2-(R1) guidelines [44] were used to validate the green reverse-phase HPTLC method of caffeine quantification.

## 2. Materials and Methods

### 2.1. Materials

The working standard of caffeine (potency: 98.8%) was procured from Sigma Aldrich (St. Louis, MO, USA). HPLC-grade solvents ethanol, methanol, and chloroform were procured from E-Merck (Darmstadt, Germany). Commercial ED (ED1–ED10) and herbal products (F1–F3) containing caffeine were procured from a local market in Al-Kharj, Saudi Arabia.

### 2.2. Chromatography and Analytical Conditions

The reverse-phase HPTLC analysis was performed using the CAMAG HPTLC instrument (CAMAG, Muttenz, Switzerland). The estimation of caffeine was performed on 10 × 20 cm glass plates precoated with reverse-phase silica gel 60 F254S plates (E-Merck, Darmstadt, Germany). The samples to the reverse-phase TLC plates were spotted as the 6 mm bands utilizing a CAMAG Automatic Sampler 4 (ATS4) applicator (CAMAG, Geneva, Switzerland). The sample applicator was fitted with CAMAG Microliter Syringe (Hamilton, Bonaduz, Switzerland). The application rate for caffeine estimation was constant at 150 nL s^−1^. The TLC plates were developed in an Automatic Developing Chamber 2 (ADC 2) (CAMAG, Muttenz, Switzerland) to a distance of 80 mm using EtOH-water (55:45, *v v*^−1^) greener mobile phase. The development chamber was saturated with vapors of EtOH-water (55:45, *v v*^−1^) for 30 min at 22 °C. Caffeine was detected using a wavelength of 275 nm at a scanning rate of 20 mm s^−1^ and slit size set to 4 × 0.45 mm. 

### 2.3. Preparation of Caffeine Standard Solutions for Calibration and Quality Control (QC) Samples

Caffeine standard (10 mg) was dissolved in 100 mL of EtOH-water (55:45, *v v*^−1^) solvent system to generate a stock solution of caffeine with a concentration of 100 μg mL^−1^. To achieve caffeine concentrations in the 50–800 ng band^−1^ range, different amounts of this stock solution were diluted further using EtOH-water (55:45, *v v*^−1^) solvent system. Caffeine solutions were spotted onto TLC plates and peak area for caffeine was measured. The caffeine calibration curve was created by plotting caffeine concentrations vs. observed peak area. In addition, three QC solutions were obtained separately for derivation of validation parameters for the HPTLC method, including low QC (LQC; 100 ng band^−1^), middle QC (MQC; 500 ng band^−1^), and high QC (HQC; 800 ng band^−1^). 

### 2.4. Sample Processing of Caffeine from Marketed ED

Diverse ingredients are present in commercial ED of caffeine. To eliminate all produced gases, the commercial ED (ED1–ED10) samples were degassed using an ultrasonic bath. All ED samples were lyophilized for five days by transferring them into lyophilizer. After the lyophilization process, the obtained dried samples were dissolved in methanol-water (25: 75 *v v*^−1^). The liquid-liquid extraction was performed using chloroform to extract caffeine. The collected chloroform fractions were dried under reduced pressure using a rotary evaporator at 40 °C. The collected samples were utilized as test solutions to assess the presence of caffeine in marketed ED.

### 2.5. Processing of Samples for the Determination of Caffeine in Marketed Herbal Products 

The average weight of ten marketed tablets from each herbal product (F1–F3) was computed. For each brand (equivalent to 10 mg of caffeine), the tablets were crushed and powdered. A quantity of each brand’s powder was extracted separately using chloroform (3 × 70 mL) for 30 min. The chloroform extracts from each sample were combined and concentrated in vacuo. The concentrate from each sample was separately reconstituted in 10 mL chloroform and stored under refrigeration till further use. Using the developed method, the acquired samples were used to quantify the caffeine contents in marketed herbal products. 

### 2.6. Validation Parameters

Using ICH-Q2-R1 guidelines [44], the developed analytical method for caffeine estimation was validated for linearity range, accuracy, precision, robustness, sensitivity, and specificity. Caffeine linearity was assessed by plotting caffeine concentrations vs. observed peak area. Caffeine linearity was determined in the 100–800 ng band^−1^ range. The system suitability was assessed using the “retardation factor (R_f_), asymmetry factor (As), and number of theoretical plates per meter (N m^−1^)”. The “R_f_, As, and N m^−1^” values were calculated using their stated formulae at MCQ (500 ng band^−1^) [45]. 

The accuracy of the suggested analytical procedure was evaluated using the percent recovery. The percent recovery of caffeine was evaluated at LQC (100 ng band^−1^), MQC (500 ng band^−1^), and HQC (800 ng band^−1^).

Caffeine was measured at LQC, MQC, and HQC on the same day to evaluate intraday precision, while, it was measured at LQC, MQC, and HQC on three consecutive days to assess intermediate precision for the developed analytical method [44]. 

To evaluate the robustness of the developed HPTLC method, slight deliberate changes in the mobile phase composition were introduced. For this purpose, mobile phase was changed to EtOH-water (57:43, *v v*^−1^) and EtOH-water (53:47, *v v*^−1^), and the changes in peak area and R_f_ values were recorded [44].

The method sensitivity was determined for limit of detection (LOD) and limit of quantification (LOQ), utilizing a standard deviation approach. The LOD and LOQ values for the developed analytical method were determined using their regular equations [44,45].

The method specificity/peak purity was assessed by comparing the R_f_ values and UV spectra of caffeine in ten different marketed ED and three different herbal products to those of standard caffeine.

### 2.7. Determination of Caffeine in Marketed ED and Herbal Products 

The caffeine peak areas of the prepared solutions of ten different marketed ED and three different herbal products were obtained on reverse-phase TLC plates. The amount of caffeine in all these samples was determined using the caffeine calibration curve for the developed method.

### 2.8. Greenness Assessment

The “AGREE metric technique” [38] was used to assess the greener profile of the developed method. The AGREE scales (in the range of 0.0–1.0) for the developed method were determined using “AGREE: The Analytical Greenness Calculator (version 0.5, Gdansk University of Technology, Gdansk, Poland, 2020)”.

## 3. Results and Discussion 

### 3.1. Method Development

Literature survey revealed a limited number of green analytical methods for the determination of caffeine. As a consequence, this investigation was designed to develop and validate a green reverse-phase HPTLC method for caffeine estimation in ten different marketed ED and three different herbal products. 

**Figure 1:**
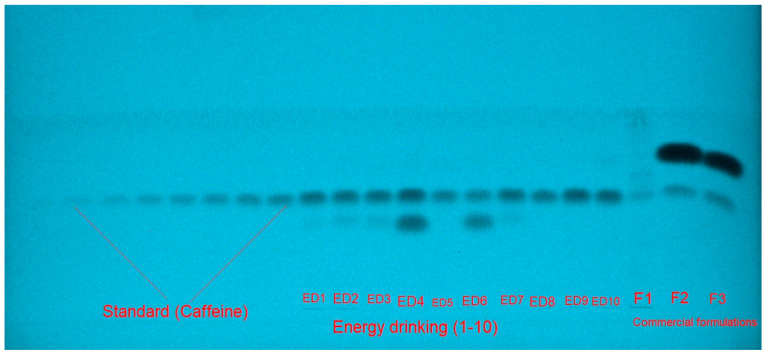
Developed plate for standard caffeine, various energy drinks (ED), and commercial formulations established using EtOH-water (55:45, *v v*^−1^) combination for the green high-performance thin-layer chromatography (HPTLC) method.

**Figure 2:**
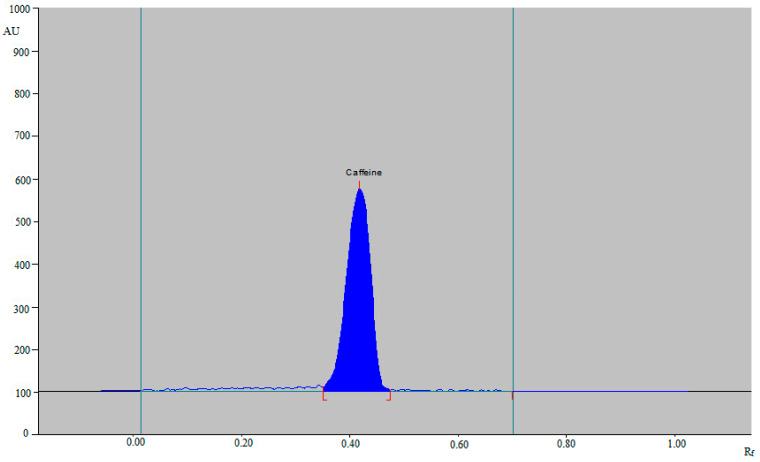
Representative chromatogram of standard caffeine obtained using the green HPTLC method.

For the estimation of caffeine in ten different marketed ED and three different herbal products, various EtOH-water concentrations within the 45–90% EtOH range were studied as the green mobile phases for method development. All of these binary solvent compositions were created in a saturated chamber (Figure 1). The results obtained showed that the EtOH-water (55:45, *v v*^−1^) solvent composition offered a well-resolved intact peak of caffeine at R_f_ = 0.42 ± 0.01 with an acceptable As value (As = 1.06 ± 0.02) (Figure 2). As a consequence, the EtOH-water (55:45, *v v*^−1^) binary solvent composition was selected as the mobile phase for caffeine estimation. The UV-spectral bands for the developed method were evaluated at absorbance mode and the greatest TLC response was found at 275 nm. 

### 3.2. Validation Parameters

The developed analytical method for caffeine estimation was validated for linearity range, accuracy, precision, robustness, sensitivity, and specificity [44]. The findings of the linearity data of the caffeine calibration curve for the developed method are included in Table 1. The caffeine calibration curve was linear in the concentration range of 50–800 ng band^−1^ for the developed method. The “determination coefficient (R^2^)” and “regression coefficient (R)” for caffeine were found to be 0.9973 and 0.9986, respectively. According to these data, there was a good linear relationship between caffeine concentration and measured TLC response.

At MQC (500 ng band^−1^), the system suitability parameters for the developed analytical test were evaluated, and the findings are given in Table 2. The “R_f_, As, and N m^−1^” values for the developed analytical test were determined to be 0.42 ± 0.01, 1.06 ± 0.02, and 5241 ± 4.58, respectively. The results showed that the developed method was suitable for estimating caffeine in ten different marketed ED and three different herbal products. 

The accuracy assessment findings for the developed method are listed in Table 3. The percent caffeine recovery for the developed method was found to be between 98.5 and 101.6 percent at three distinct QC levels. The accuracy of the developed method for caffeine estimation in ten different marketed ED and three distinct herbal products was demonstrated by these percent caffeine recoveries.

The precision for the developed method was measured as the percent of the coefficient of variance (% CV) and the findings are listed in Table 4. The results revealed the precision of the developed method for caffeine estimation in ten different marketed ED and three different herbal products.

The data from the robustness study for the developed method are listed in Table 5. The percent CVs for the robustness analysis for the developed method were 0.65–0.77%. The R_f_ values of caffeine were found to be 0.41–0.43. The minor deviations in the R_f_ values of caffeine and lower % CVs revealed the robustness of the developed method for caffeine estimation in ten ED and three herbal products. 

The sensitivity of the developed method was measured as “LOD and LOQ” and the findings are presented in Table 1. The “LOD and LOQ” for the developed method were determined to be 16.87 ± 0.45 and 50.61 ± 1.35 ng band^−1^, respectively, for caffeine estimation. These values of “LOD and LOQ” for the developed method revealed the sensitivity for the estimation of low levels of caffeine in ten different marketed ED and three different herbal products. 

**Figure 3:**
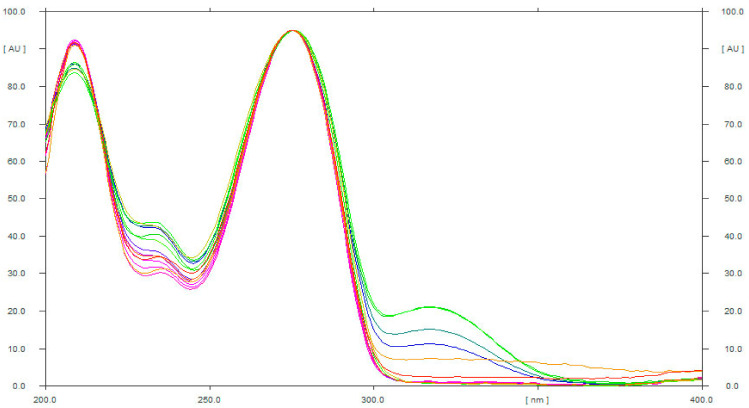
UV absorption spectra of standard caffeine, different ED, and various commercial formulations, superimposed.

The peak purity and specificity of the developed method was determined by comparing the UV spectra of caffeine (275 nm) in ten different marketed ED and three different herbal products with that of standard caffeine. Figure 3 presents the UV spectra of standard caffeine and caffeine in ten different marketed ED and three different herbal products, superimposed. The identical UV spectra, R_f_ values, and detection wavelength of caffeine in standard caffeine, ten commercial ED, and three herbal products demonstrated the peak purity and specificity of the developed method.

### 3.3. Determination of Caffeine in Marketed ED and Herbal Products 

**Figure 4:**
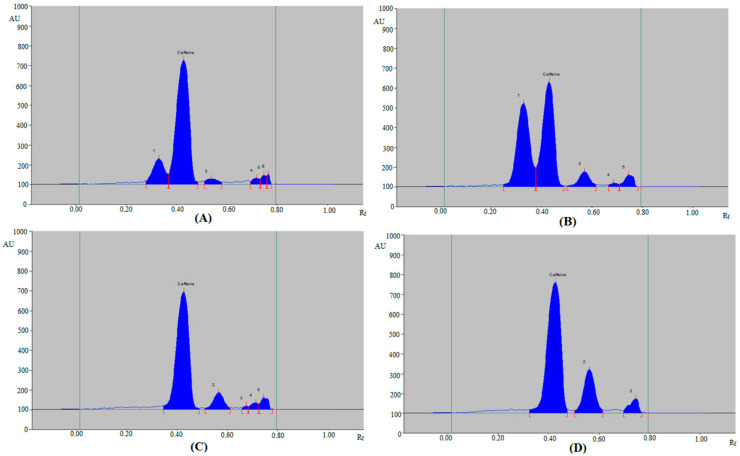
Representative chromatograms of caffeine in (**A**) ED-3, (**B**) ED-6, (**C**) ED-8, and (**D**) ED-10.

**Figure 5:**
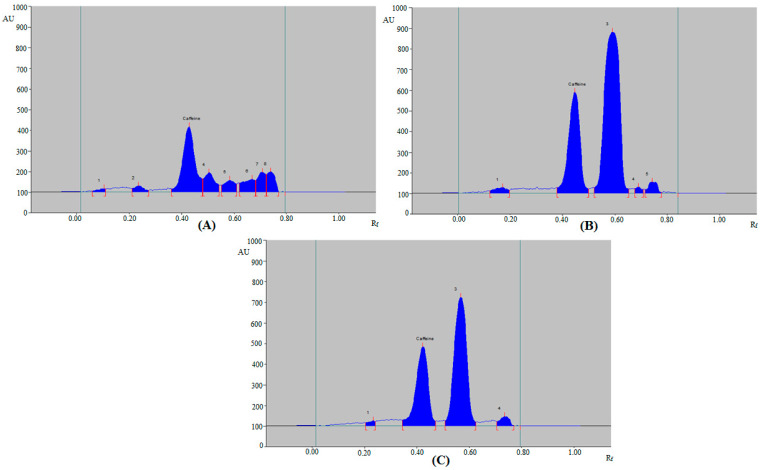
Representative chromatograms of caffeine in (**A**) marketed formulation F1, (**B**) marketed formulation F2, and (**C**) marketed formulation F3.

The validated method was applied to estimate caffeine content in ten marketed ED and three herbal products. By comparing the single TLC spot at R_f_ = 0.42 ± 0.01 for caffeine in ED and herbal products with those of standard caffeine, the HPTLC peak of caffeine from ten ED and three herbal products was verified. The representative densitograms of caffeine in some ED, such as ED3, ED6, ED8, and ED10 are included in Figure 4, which presented concordant peaks of caffeine with that of standard caffeine. Similar chromatograms were also recorded for other ED, such as ED1, ED2, ED4, ED5, ED7, and ED9 (Figure not shown). Furthermore, five, four, four, and two additional peaks were also detected in ED3 (Figure 4A), ED6 (Figure 4B), ED8 (Figure 4C), and ED10 (Figure 4D), respectively. The densitograms of caffeine in herbal products F1–F3 are included in Figure 5, which also presented the caffeine peak. Additionally, seven, four, and three peaks were also detected in herbal products F1 (Figure 5A), F2 (Figure 5B), and F3 (Figure 5C), respectively. The existence of extra peaks in various ED and herbal products indicated that the developed HPTLC method might be used to estimate caffeine in the presence of impurities/different substances. The caffeine content of ten different ED and three herbal products was estimated using caffeine calibration curve and the findings are summarized in Table 6. The amount of caffeine in the ED (ED1–ED10) was recorded in the range of 21.02–37.52 mg 100 mL^−1^. However, the amount of caffeine in the herbal products (F1–F3) was 10.63–20.30 mg 100 mL^−1^. The caffeine content of the products was expressed as a percentage of the label claim for each product as recorded in Table 6. These results demonstrated the suitability of the developed HPTLC method in determining caffeine content in marketed ED and herbal products. 

### 3.4. Greenness Assessment

**Figure 6:**
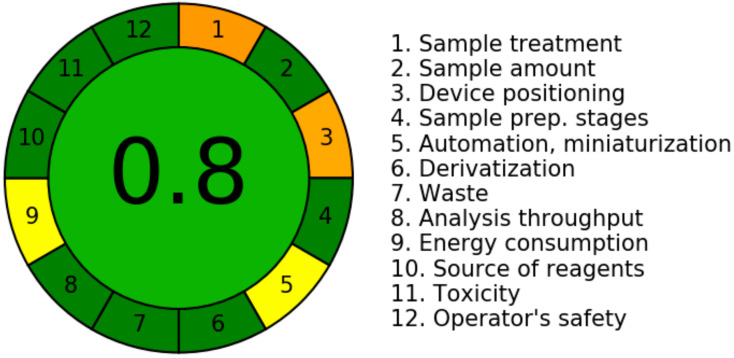
Representative diagram for AGREE scale for the green HPTLC method estimated utilizing “AGREE: The Analytical Greenness Calculator”.

Despite the fact that several approaches for determining a greener scale of analytical methods have been documented [34,35,36,37,38,39], only the “AGREE methodology” [38] employs all twelve GAC principles. As a result, the “AGREE Calculator” was utilized to determine the greenness scale of the developed HPTLC method. The overall AGREE score for the developed method is shown in Figure 6. The overall AGREE score for the developed method was calculated to be 0.80, indicating that the developed method for caffeine estimation is exceptionally green. 

## 4. Conclusions

The objective of this study is to develop and validate a reverse-phase HPTLC method for estimating caffeine in ten different commercial ED and three different herbal products. The developed HPTLC method was validated according to ICH guidelines. The developed method was linear, rapid, accurate, precise, robust, sensitive, specific, and green for caffeine estimation. The AGREE scale for the developed method showed its excellent green profile for caffeine estimation. The developed reverse-phase HPTLC method was found to be suitable for caffeine estimation in ten different marketed ED and three different marketed herbal products. These results and data indicated that the green reverse-phase HPTLC method can be successfully applied for the determination of caffeine in the wide range of commercial products having caffeine as one of the active ingredients.

## Figures and Tables

**Table 1:** Results for the regression analysis of caffeine for the green high-performance thin-layer chromatography (HPTLC) method (mean ± SD; *n* = 6).

Parameters	Values
Linearity range (ng band^−1^)	50–800
Regression equation	y = 28.429x + 813.89
R^2^	0.9973
R	0.9986
SE of slope	0.35
SE of intercept	2.42
95% CI of slope	26.90–29.95
95% CI of intercept	803.45–824.32
LOD ± SD (ng band^−1^)	16.87 ± 0.45
LOQ ± SD (ng band^−1^)	50.61 ± 1.35

R^2^: determination coefficient; R: regression coefficient; SE: standard error; CI: confidence interval; LOD: limit of detection; LOQ: limit of quantification.

**Table 2:** System suitability parameters of caffeine for the green HPTLC method (mean ± SD; *n* = 3).

Parameters	Green HPTLC Method
R_f_	0.42 ± 0.01
As	1.06 ± 0.02
N m^−1^	5241 ± 4.58

R_f_: retardation factor; As: asymmetry factor; N m^−1^: number of theoretical plates per meter.

**Table 3:** Accuracy assessment findings of caffeine for the green HPTLC method (mean ± SD; *n* = 6).

Conc. (ng Band−1)	Conc. Found (ng Band−1) ± SD	Recovery (%)	CV (%)
100	98.52 ± 1.36	98.5	1.38
500	508.23 ± 3.54	101.6	0.69
800	788.36 ± 6.94	98.5	0.88

**Table 4:** Precision assessment findings of caffeine for the green HPTLC method (mean ± SD; *n* = 6).

Conc. (ng Band^−1^)	Intraday Precision			Interday Precision		
	Conc. Found (ng Band^−1^) ± SD	Standard Error	CV (%)	Conc. Found (ng Band^−1^) ± SD	Standard Error	CV (%)
100	101.45 ± 0.84	0.34	0.82	99.21 ± 0.92	0.37	0.92
500	494.31 ± 3.11	1.26	0.62	491.23 ± 3.53	1.44	0.71
800	792.34 ± 4.74	1.93	0.59	806.32 ± 5.15	2.10	0.63

**Table 5:** Robustness assessment of findings of caffeine for the green HPTLC method (mean ± SD; *n* = 6).

Conc. (ng Band^−1^)	Mobile Phase Composition (EtOH-Water)			Results		
	Original	Used		Conc. (ng Band^−1^) ± SD	CV (%)	R_f_
		57:43	+2.0	492.54 ± 3.22	0.65	0.41
500	55:45	55:45	0.0	504.51 ± 3.66	0.72	0.42
		53:47	−2.0	511.21 ± 3.97	0.77	0.43

**Table 6:** Determination of caffeine in different energy drinks (ED) and various commercial formulations using the green HPTLC method (mean *±* SD; *n* = 3).

Samples	Label Content of Caffeine (mg 100 mL^−1^)	Caffeine Found (mg 100 mL^−1^)	Percent of Label Amount
ED1	30	30.52 ± 1.01	98.2
ED2	30	29.19 ± 1.00	102.7
ED3	32	31.21 ± 1.03	102.5
ED4	32	32.89 ± 1.04	97.2
ED5	30	28.08 ± 1.01	106.8
ED6	20	21.02 ± 0.80	95.1
ED7	32	31.88 ± 2.02	100.3
ED8	30	29.98 ± 1.01	100.0
ED9	35	37.52 ± 2.01	93.2
ED10	35	35.93 ± 2.04	97.3
F1	10	10.63 ± 0.50	94.0
F2	20	20.30 ± 0.60	98.4
F3	15	15.92 ± 0.65	94.2

## Data Availability

Not applicable.

## References

[1] Shakeel F., Ramadan W. (2010). Transdermal delivery of anticancer drug caffeine from water-in-oil nanoemulsions. Colloids Surf. B.

[2] Abourashed E.A., Mossa J.S. (2004). HPTLC determination of caffeine in stimulant herbal products and power drinks. J. Pharm. Biomed. Anal..

[3] Faudone G., Arifi S., Merk D. (2021). The medicinal chemistry of caffeine. J. Med. Chem..

[4] Nimbhorkar R., Rasane P., Singh J. (2021). Caffeine alternatives: Seraching a herbal solution. Pharm. Innov. J..

[5] Rahimi M., Khorshidi N., Heydari R. (2020). Simultaneous determination of paracetamol and caffeine in aqueous samples by ultrasound-assisted emulsification microextraction coupled with high-performance liquid chromatography. Sep. Sci. Plus.

[6] Tautua A., Bamidele W., Diepreye E.R.E. (2014). Ultra-violet spectrophotometric determination of caffeine in soft and energy drinks available in Yenagoa, Nigeria. Adv. J. Food Sci. Technol..

[7] Khalid A., Ahmad S., Raza H., Batool M., Lodhi R.K., Ain Q.T., Naseer F. (2016). Determination of caffeine in soft and energy drinks available in market by using UV/visible specrtophotometer. Fam. Med. Med. Sci. Res..

[8] Ogunneye A.L., Banjoko O.O., Gbadamosi M.R., Falegbe O.H., Moberuagba K.H., Badejo O.A. (2020). Spectrophotometric determination of caffeine and vitamin B6 in selected beverages, energy/soft drinks and herbal products. Niger. J. Basic Appl. Sci..

[9] Deasi S. (2020). Estimation of caffeine content from soft and energy drinks obtained from regional markets by UV spectroscopy and TLC. Int. J. Sci. Dev. Res..

[10] Vuletic N., Bardic L., Odzak R. (2021). Spectrophotometric determination of caffeine content in the selection of teas, soft and energy drinks available on the Croatian market. Food Res..

[11] Kaurav M.G., Kihunyu J.N., Kathenyal N.M., Wangai L.N., Kariuki D., Kibet R.H. (2010). Determination of caffeine content in non-alcoholic beverages and energy drinks using HPLC-UV method. Afr. J. Drug Alcohol Stud..

[12] Nour Y., Trandafir I., Ionica M.E. (2010). Chromatographic determination of caffeine contents in soft and energy drinks available on the Romanian market. St. Cerc. St. CICBIA.

[13] Ali M.M., Eisa M., Taha M.I., Zakaria B.A., Elbashir A.A. (2012). Determination of caffeine in some Sudanese beverages by high performance liquid chromatography. Pak. J. Nutr..

[14] Rudolph E., Farbinger A., Konig J. (2012). Determination of caffeine contents of various food items within the Austrian market and validation of a caffeine assessment tool (CAT). Food Addit. Contam. Part A.

[15] Al-Othman Z.A., Aqel A., Alharbi M.K.E., Badjah-Hadj-Ahmed A.Y., Al-Warthan A.A. (2012). Fast chromatographic determination of caffeine in food using a capillary hexyl methacrylate monolithic column. Food Chem..

[16] Gliszczynska-Swiglo A., Rybicka I. (2015). Simultaneous determination of caffeine and water-soluble vitamins in energy drinks by HPLC with photodiode array and fluorescence detection. Food Anal. Methods.

[17] Rai K.P., Rai H.B., Dahal S., Chaudhary S., Shrestha S. (2016). Determination of caffeine and taurine in energy drinks by HPLC-UV. J. Food Sci. Technol. Nepal.

[18] Mirza J., Sultana M., Esrafil M., Akter S., Alam M.J., Khan M.S.H., Zubair M.A. (2021). High-performance liquid chromatographic method for quantitative determination of caffeine in different soft and energy drinks available in Bangladesh. Curr. Res. Nutr. Food Sci..

[19] Medina I.Y., Rodriguez D.C., Parra J.W. (2020). Analysis of caffeine in energy drinks by ultra-fast liquid chromatography. J. Phys. Conf. Ser..

[20] Lee M.S., Huong N.L., Hoang N.H., Shresthan A., Park J.W. (2014). Ultra-performance liquid chromatography with electrospray ionization tandem mass spectrometry for the determination of caffeine in energy drinks. Anal. Lett..

[21] Mohammed S.G., Al-Hashimi A.G., Al-Hussainy K.S. (2012). Determination of caffeine and trace mineral contents in soft and energy drinks available in Basrah markets. Pak. J. Nutr..

[22] Torres J.L.T., Hiley S.L., Lorimor S.P., Rhoad J.S., Caldwell B.D., Zweerink G.L., Ducey M. (2015). Separation of caffeine from beverages and analysis using thin-layer chromatography and gas chromatography-mass spectrometry. J. Chem. Educ..

[23] Al-Bratty M., Alhazmi H.A., Rehman Z.U., Javed S.A., Ahsan W., Najmi A., Khuwaja G., Makeen H.A., Khalid A. (2020). Determination of caffeine content in commercial energy beverages available in Saudi Arabian market by gas chromatography-mass spectrometric analysis. J. Spectrosc..

[24] Tavallali H., Zareiyan S.F.J., Naghian M. (2011). An efficient and simultaneous analysis of caffeine and paracetamol in pharmaceutical formulations using TLC with a fluorescence plate reader. J. AOAC Int..

[25] Riswanto F.D.O., Lukitaningsih R.R.E., Martono S. (2015). Analytical method validation and determination of pyridoxine, nicotinamide, and caffeine, in energy drinks using thin layer chromatography-densitometry. Indones. J. Chem..

[26] Oellig C., Schunck J., Schwack W. (2018). Determination of caffeine, theobromine and theophylline in mate beer and mate soft drinks by high-performance thin-layer chromatography. J. Chromatogr. A.

[27] Armenta S., Garrigues S., de la Guardia M. (2005). Solid-phase FT-Raman determination of caffeine in energy drinks. Anal. Chim. Acta.

[28] Grant D.C., Helleur R.J. (2008). Simultaneous analysis of vitamins and caffeine in energy drinks by surfactant-mediated matrix-assisted laser desorption/ionization. Anal. Bioanal. Chem..

[29] Liotta E., Gottardo S., Seri C., Rimondo C., Miksik I., Serpelloni G., Tagliaro F. (2012). Rapid analysis of caffeine in “smart drugs” and “energy drinks” by microemulsion electrokinetic chromatography (MEEKC). Forensic Sci. Int..

[30] Vochyanova B., Opekar F., Tuma P. (2014). Simultaneous and rapid determination of caffeine and taurine in energy drinks by MEKC in a short capillary with dual contactless conductivity/photometry detection. Electrophoresis.

[31] Alam P., Shakeel F., Ali A., Alqarni M.H., Foudah A.I., Aljarba T.M., Alkholifi F.K., Alshehri S., Ghoneim M.M., Ali A. (2022). Simultaneous determination of caffeine and paracetamol in commercial formulations using greener normal-phase and reversed-phase HPTLC methods: A contrast of validation parameters. Molecules.

[32] Ibrahim F.A., Elmansi H., Fathy M.E. (2019). Green RP-HPLC method for simultaneous determination of moxifloxacin combinations: Investigation of the greenness for the proposed method. Microchem. J..

[33] Abou-Taleb N.H., Al-Enany N.M., El-Sherbiny D.T., El-Subbagh H.I. (2020). Digitally enhanced thin layer chromatography for simultaneous determination of norfloxacin tinidazole with the aid of Taguchi orthogonal array and desirability function approach: Greenness assessment by analytical eco-scale. J. Sep. Sci..

[34] Foudah A.I., Shakeel F., Alqarni M.H., Ali A., Alshehri S., Ghoneim M.M., Alam P. (2022). Determination of thymol in commercial formulations, essential oils, traditional, and ultrasound-based extracts of *Thymus vulgaris* and *Origanum vulgare* using a greener HPTLC approach. Molecules.

[35] Alqarni M.H., Shakeel F., Mahdi W.A., Foudah A.I., Aljarba T.M., Alshehri S., Ghoneim M.M., Alam P. (2022). A greener stability-indicating high-performance thin-layer chromatography approach for the estimation of topiramate. Materials.

[36] Abdelrahman M.M., Abdelwahab N.S., Hegazy M.A., Fares M.Y., El-Sayed G.M. (2020). Determination of the abused intravenously administered madness drops (tropicamide) by liquid chromatography in rat plasma; an application to pharmacokinetic study and greenness profile assessment. Microchem. J..

[37] Duan X., Liu X., Dong Y., Yang J., Zhang J., He S., Yang F., Wang Z., Dong Y. (2020). A green HPLC method for determination of nine sulfonamides in milk and beef, and its greenness assessment with analytical eco-scale and greenness profile. J. AOAC Int..

[38] Pena-Pereira F., Wojnowski W., Tobiszewski M. (2020). AGREE-Analytical GREEnness metric approach and software. Anal. Chem..

[39] Nowak P.M., Koscielniak P. (2019). What color is your method? Adaptation of the RGB additive color model to analytical method evaluation. Anal. Chem..

[40] Karmaker R., Sinha D., Sinha U.B. (2021). Rationalizing between the efficiency and greenness of solvents—A computational study of their influence on TBATB. Sustain. Chem. Pharm..

[41] Kim D., Nunes S.P. (2021). Green solvents for membrane manufacture: Recent trends and perspectives. Curr. Opin. Green Sustain. Chem..

[42] Byrne F.P., Jin S., Paggiola G., Petchey T.H.M., Clark J.H., Farmer T.J., Hunt A.J., McElroy C.R., Sherwood J. (2016). Tools and techniques for solvent selection: Green solvent selection guides. Sustain. Chem. Processes.

[43] Foudah A.I., Shakeel F., Alqarni M.H., Ross S.A., Salkini M.A., Alam P. (2022). Green NP-HPTLC and green RP-HPTLC methods for the determination of thymoquinone: A contrast of validation parameters and greenness assessment. Phytochem. Anal..

[44] Q2 (R1) validation of analytical procedures–text and methodology. Proceedings of the International Conference on Harmonization (ICH).

[45] Foudah A.I., Shakeel F., Alqarni M.H., Alam P. (2021). A rapid and sensitive stability-indicating RP-HPTLC method for the quantitation of flibanserin compared to green NP-HPTLC method: Validation studies and greenness assessment. Microchem. J..

